# Main Chain Polysulfoxides as Active ‘Stealth’ Polymers with Additional Antioxidant and Anti-Inflammatory Behaviour

**DOI:** 10.3390/ijms20184583

**Published:** 2019-09-17

**Authors:** Farah El Mohtadi, Richard d’Arcy, Xiaoye Yang, Zulfiye Yesim Turhan, Aws Alshamsan, Nicola Tirelli

**Affiliations:** 1 Division of Pharmacy and Optometry, School of Health Sciences, University of Manchester, Oxford Road, Manchester M13 9PT, UK; Farah.elmohtadi@manchester.ac.uk (F.E.M.); yangxiaoyechn@outlook.com (X.Y.); zulfiyeyesim.turhan@manchester.ac.uk (Z.Y.T.); 2 Laboratory for Polymers and Biomaterials, Fondazione Istituto Italiano di Tecnologia, 16163 Genova, Italy; 3 Department of Pharmaceutics, College of Pharmacy, Shandong University, Jinan 250012, China; 4 Department of Pharmaceutics, College of Pharmacy, King Saud University, P.O. Box 2457, Riyadh 11451, Saudi Arabia; aalshamsan@ksu.edu.sa; 5 Nanobiotechnology Unit, College of Pharmacy, King Saud University, P.O. Box 2457, Riyadh 11451, Saudi Arabia

**Keywords:** biocompatibility, bioinertness, oxidants, therapeutic polymers, responsive polymers, polysulfides

## Abstract

We present the evaluation of a sulfoxide-based polymer (poly(propylene sulfoxide), PPSO) as a potential ‘stealth’ macromolecule, and at the same time as a pharmacologically active (anti-inflammatory/anti-oxidant) material. The combination of these two concepts may at first seem peculiar since the gold standard polymer in biomaterials and drug delivery, poly(ethylene glycol) (PEG), is ‘stealth’ due to its chemical and biological inertness, which makes it hardly biologically active. Polysulfoxides, on the contrary, may couple a substantial inertness towards biomolecules under homeostatic conditions, with the possibility to scavenge reactive oxygen species (ROS) associated to inflammation. Polysulfoxides, therefore, are rather uniquely, ‘active’ ‘stealth’ polymers. Here, we describe the synthesis of PPSO through controlled oxidation of poly(propylene sulfide) (PPS), which on its turn was obtained via anionic ring-opening polymerization. In vitro, PPSO was characterized by a low toxicity (IC50 ~7 mg/mL at 24 h on human dermal fibroblasts) and a level of complement activation (in human plasma) and macrophage uptake slightly lower than PEG of a similar size. Importantly, and differently from PEG, on LPS-activated macrophages, PPSO showed a strong and dose-dependent ROS (hydrogen peroxide and hypochlorite)-scavenging activity, which resulted in a corresponding reduction of cytokine production.

## 1. Introduction

**Scheme 1:**
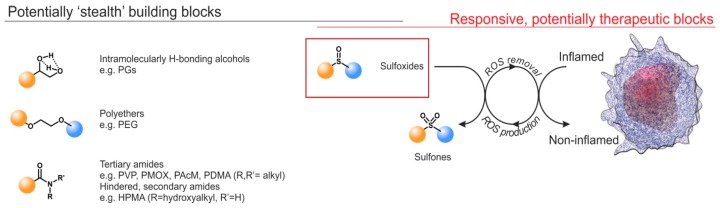
Polysulfoxides can present features of ‘stealth’ materials (on the left hand of the scheme; common features: lack of nucleophilic or H-bond donating character, hydrophilicity) and of responsive structures (on the right hand of the scheme: OX-response through conversion of the sulfoxides to sulfones). Their potential for scavenging reactive oxygen species (ROS) can result in an overall anti-inflammatory activity.

For decades, poly(ethylene glycol) (PEG) has been considered the golden standard of synthetic macromolecules in biomedical applications, be it in the preparation of carriers for drug delivery (including several marketed or FDA-approved products [1,2]), proteins with reduced immunogenicity, [3,4], or matrices for tissue engineering/regenerative medicine [5]. PEG owes its widespread use to a combination of low toxicity [6] and ‘stealth’ character, which can be summarized as a prolonged circulation in body fluids and reduced clearance, as a result of negligible protein adsorption (opsonization) [4,7] and low activation of immune system components [8]. Indeed, an increase in hydrodynamic size upon PEGylation usually contributes to an overall longer circulation. PEG is not totally unique though: A number of polymers have been proposed as alternative stealth polymers, e.g., poly[N-(2-hydroxypropyl) methacrylamide](HPMA) [9,10,11], poly(vinylpyrrolidone)(PVP) [12,13], poly(2-methyl-2-oxazoline) (PMOX); [14,15], poly(N-acryloyl morpholine)(PAcM) [16,17], poly(N,N-dimethylacrylamide)(PDMA) [18], and polyglycerols [19]. The common feature in their macromolecular structure is the presence of very hydrophilic groups, which are poor nucleophiles and/or H-bond acceptors but not donors (Scheme 1, left).

‘Stealth’ materials have their drawbacks too, chiefly of an immunological nature, such as the accelerated blood clearance (ABC) [20,21] after repeated injections of PEGylated materials. This phenomenon is predominantly ascribed to the production anti-PEG IgM antibodies [22,23], and may also link to the significant complement activation that PEG and PEG-containing structures can elicit in vivo [24,25]. IgMs bind to PEGylated carriers upon their second or later administration, and lead to their uptake by Kupffer cells in the liver [20,26], thereby competing and eventually overcoming the accumulation in tumours, the so-called enhanced permeation and retention (EPR) effect of PEGylated carriers [27]. Importantly, ABC is not observed only with PEG; for example, PMOX has recently been found to display an ABC effect macroscopically similar to that of PEG [28].

Another historical limit of ‘stealth’ drug delivery systems is that, as obvious as it may sound, biological inertness typically also means chemically and biologically inactive/unresponsive; for example, the presence of PEG—by virtue of its biological inertness—may reduce or slow down the accumulation of a carrier in a tissue or its endosomal escape (sometimes known as the “PEG dilemma”) [7].

In this study, we focus on sulfoxides as building blocks of ‘stealth’ structures. Polymers based on sulfoxides are known for their very low cytotoxicity [29,30,31], and the idea to use them in a biological environment is not new. In the mid 1970s, Helmut Ringsdorf already saw the potential advantages of sulfoxide-containing polymers and studied side-chain sulfoxide polyacrylates (in particular poly(2-(methylsulfinyl)ethyl acrylate), PMSEA) as biocompatible and bioresorbable penetration enhancers [32]. PMSEA was recently proposed again by Matyjaszewski with more modern syntheses based on Atom Transfer Radical Polymerization (ATRP) [29], electrochemically mediated ATRP (eATRP) [33], and surface-initiated ATRP [34]. PMSEA has often been shown to behave similarly to poly(PEG acrylate), with the possible advantage of a lower steric hindrance; for example, their block copolymers with fluorine-containing monomers have a comparable MRI performance (slightly more intense signals for PMSEA) [35], and cationic hyperbranched constructs have similar transfection efficiency (but better polyplex formation for PMSEA) [36].

PMSEA is also occasionally referred to as poly(DMSO) [35] due its dimethyl sulfoxide (DMSO)-like side chain (-CH_2_S(=O)CH_3_). DMSO is indeed in itself one of the most benign/low toxicity organic solvents, being regularly used as a cryoprotective agent for cells and above all as a pharmaceutical excipient. Originally intended almost exclusively for the transdermal (topical) route, it is now increasingly employed in several parenteral and oral formulations [37]. Although toxic at high concentrations [38], at low concentrations, DMSO has a significant anti-inflammatory behaviour, e.g., reducing the lymphocyte activation [39], inhibiting M1 or M2 macrophage polarization, and ameliorating rheumatoid arthritis symptoms in mice [40]. These effects are likely due to DMSO’s antioxidant (hydroxyl radical sequestering) activity of DMSO [41].

Our group has a long-running activity on oxidation-sensitive polymers [42]. We have specifically focused on polysulfides, employing them as reactive oxygen species (ROS)-scavengers, and as a result as anti-inflammatory agents [43,44]. In previous studies, we showed how the oxidation of thioethers can either stop at the level of sulfoxides or proceed to sulfones, depending on the conditions of oxidation and the nature of oxidant [30,45]. Here, we focus on the selective oxidation of poly(propylene sulfide) (PPS) to poly(propylene sulfoxide) (PPSO). Specifically, we present its evaluation as both a potential ‘stealth’ and an anti-inflammatory material, thereby condensing two apparently disjointed functions into the same macromolecular structure.

## 2. Results and Discussion

### 2.1. Polymer Synthesis

**Figure 1:**
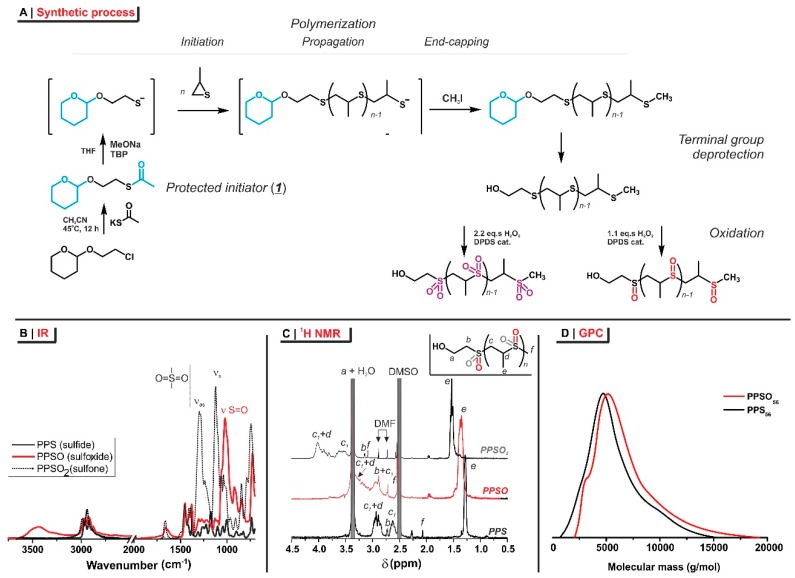
(**A**) Synthetic process for the preparation of α-hydroxy-ω-methyl-poly(propylene sulfoxide), and of the corresponding sulfone-containing (further oxidized) polymer. Diphenyl diselenide (DPDS) was used as a catalyst (at ~0.025 equiv.s per thioether) in the final oxidation reactions. In blue, the protected groups present in compound ***1***, which provides a thiolate to initiate the polymerization and a primary alcohol in the final polymer. (**B**) FT-IR spectra of PPS_56_ 24 h after oxidation with 1.1 equiv.s of H_2_O_2_ (PPSO) or 2.2 equiv.s (PPSO_2_), with the indication of bands diagnostic for the oxidized groups. The spectra are normalized to the maximum of their ν_as_ CH_3_ band at ~2960 cm^−1^. Please note that the band above 3000 cm^−1^ in PPSO’s spectrum shows the presence of water due to the hygroscopic nature of the polymer. (**C**) ^1^H NMR spectra (in *d6*-DMSO) of PPS, PPSO, and PPSO_2_. Letters correspond to the assignments labelled on the structure in the inset. (**D**) Triple detection GPC traces of PPS and PPSO (in DMF with 0.1% LiBr).

The anionic ring-opening polymerization (ROP) of propylene sulfide (PS) was initiated by a thiolate generated in situ through the deprotection of a thioacetate, in the presence of the reducing agent, tributylphosphine (Figure 1A); this combination minimizes the presence of disulfides, which act as chain transfer agents in episulfide polymerization [46], preventing them therefore from compromising the identity of the terminal groups of the final polymers, which would in turn increase their molecular weight dispersity. The polymerization was terminated by reacting the thiolate-terminated polymer with iodomethane, an end-capper previously employed by our group also for the determination of the instantaneous composition of copolysulfides and the determination of comonomeric reactivity ratios [47]. The initiator contained a second protected chemical function, a tetrahydropyranyl ether. After polymerization, the cleavage of this acetal with an acidic resin liberated a terminal primary alcohol, thereby yielding a heteroterminated polysulfide (α-hydroxy-ω-methyl-PPS, hereafter referred to as mPPSO-OH for homogeneity of nomenclature with mPEG-OH). The two different termini allowed for the determination of the polymer size ($\bar{M_{n}}$) via ^1^H NMR, which rather closely confirmed the data from GPC analysis (Table 1), as well as the efficiency of end-capping. Please note that termination and initiation groups were chosen to keep the polymers as similar as possible to the 5 kDa mPEG-OH.

The oxidation of PPS was performed using near stoichiometric amounts of H_2_O_2_ combined with catalytic amounts of DPDS; the latter has been recently introduced as a catalyst for the selective oxidation of thioethers to sulfoxides in organic solvents using stoichiometric amounts of a urea/H_2_O_2_ complex [48]. In comparison to this literature, we found that the oxidation proceeded equally well adding aqueous (30%) H_2_O_2_ to a DMF solution of PPS in the absence of urea. We also saw that the process is selective towards sulfoxides only on the basis of the amount of oxidant added: With 1.1 equiv.s of H_2_O_2_, a well-characterized PPSO is obtained (Figure 1B,C), essentially with the chain length as the parent PPS (Figure 1D). However, with 2.2 equiv.s of H_2_O_2_, a polymer was obtained (PPSO_2_) where sulfoxides were clearly further oxidized to sulfones: The PPSO_2_ IR spectrum showed the well-recognizable asymmetric (ν_as_ SO_2_) and symmetric (ν_s_ SO_2_) stretching bands of sulfones at 1296 and 1121 cm^−1^ replacing that of sulfoxides (ν S=O) at ~1017 cm^−1^ (Figure 1B), and also ^1^H NMR showed a clear downfield effect of the broad bands of a still polymeric structure (Figure 1C). It is, however, possible that PPSO_2_ has undergone significant chain cleavage: It’s very limited solubility—only in DMSO or basified water—which also prevents the usual GPC analysis—and an IR band at 844 cm^−1^ possibly ascribed to ν S-O vibration may indicate the presence of sulfenic acids. The latter are necessarily terminal groups, whose formation is consistent with the easy fragmentation (low ceiling temperature) of macromolecules based on aliphatic sulfones [30]. Due to the more difficult characterization of PPSO_2_, and to its likely low stability, in the rest of the study, we only focused on PPSO.

### 2.2. Potentially “Stealth” Character and Anti-Inflammatory Properties

**Figure 2:**
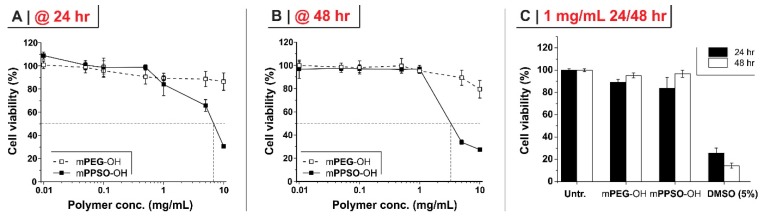
(**A**) The mitochondrial reductase activity (MTS assay) was used as a measure of the viability of HDFn, when cultured for 24 h in the presence of mPPSO-OH or the analogous mPEG-OH. Horizontal and vertical dashed lines allow the individuation of the IC50 as their intersection point (*n* = 3). (**B**) As in A, for 48 h. (**C**) Visual comparison of HDFn viability after exposure to 1 mg/mL mPPSO-OH and mPEG-OH for 24 (black bars) and 48 h (white bars), also reporting 5% DMSO as a positive control.

**Figure 3:**
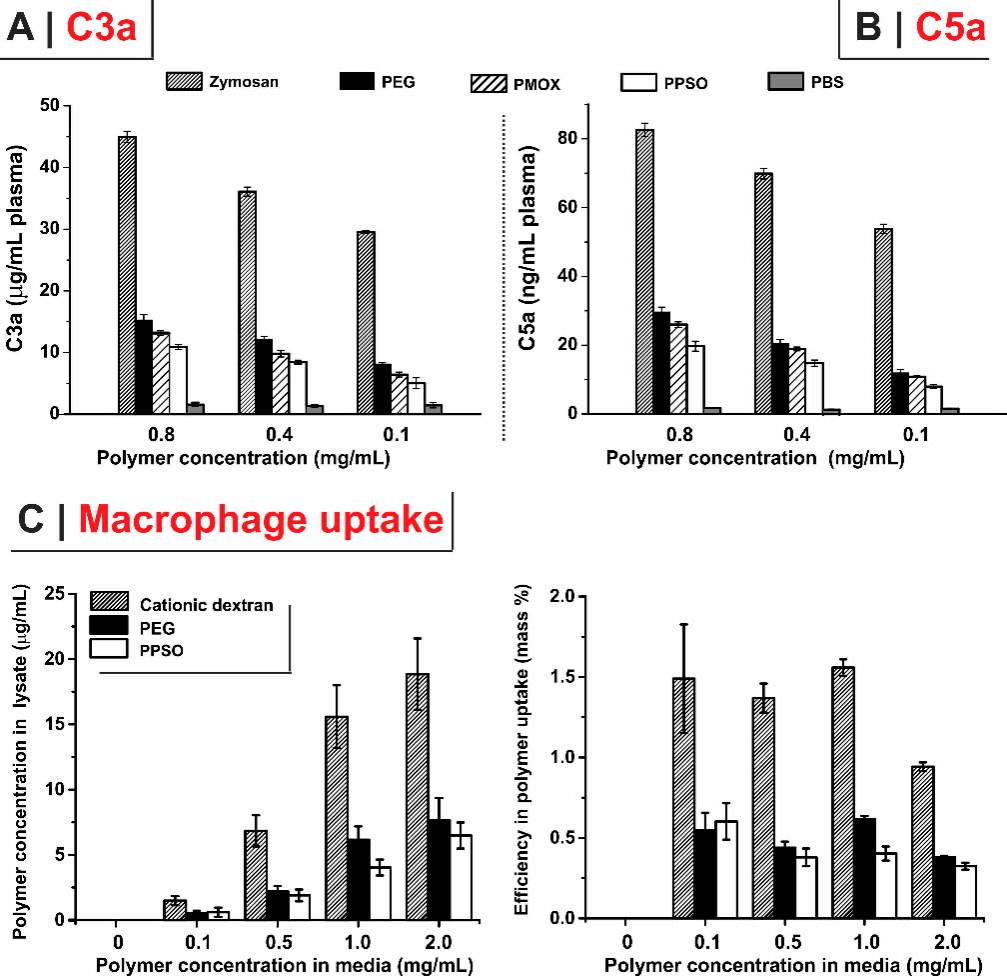
(**A**) ELISA-based assessment of the production of C3a complement fragment upon incubation of human plasma with zymosan (positive control), PEG, PMOX, PPSO, and PBS (negative control) at three different concentrations (*n* = 3). (**B**) As in A for the complement fragment C5a (right), in human plasma. (**C**) Uptake of fluorescein-labelled PEG and PPSO (the terminal OH groups were converted to amines and reacted with FITC) and FITC-labelled cationic dextran after 24 h incubation in RAW264.7 macrophages activated with 500 ng/mL LPS. The left graph reports the concentration of the polymers in 100 µL of cell lysates, the right graph normalizes the amount of polymer found in lysates (concentration*volume) against the total amount of polymer cells they were incubated with; this provides a measure of the efficiency of uptake (*n* = 3). The polymer uptake in untreated RAW264.7 macrophages is provided in Supplementary Materials, Figure 2SI.

Firstly, the PPSO backbone appeared to be characterized by a low toxicity. mPPSO-OH showed a very low capacity to reduce the viability of HDFn (IC50 ~7 mg/mL at 24 h (Figure 2A) and ~3 mg/mL at 48 h (Figure 3A) using the MTS assay). mPPSO-OH does appear to be more toxic than mPEG-OH, however, only at the very high end of the concentration range usable for in vivo applications that most commonly is well below 1 mg/mL (consider that either protein or polymer conjugates are injected to doses ranging between from µg/kg to a few mg/kg [49,50]). Indeed, at (and up to) 1 mg/mL, the two polymers are virtually indistinguishable (Figure 2C). The effect of PPSO on viability is likely ascribed to its capacity to scavenge ROS, like its parent polymer PPS [44]; small amounts of ROS are essential to many cellular processes [51] and high doses of PPSO may indeed scavenge them to less-than-homeostatic levels.

In a preliminary assessment of possible foreign-body responses, mPPSO-OH behaved similarly to ‘stealth’ polymers, such as PEG and PMOX. The complement pathway, being part of the innate immune system, is a critical defence mechanism whose activation allows recognition and elimination of potentially harmful foreign pathogens. Complement activation is therefore a major factor in the assessment of the biocompatibility of a polymer. For example, materials, such as regenerated cellulose [52], poly(hydroxyethyl methacrylate) (pHEMA) [53], and plasticized PVC [54], and most commonly polymers that are either strongly hydrophobic or displaying nucleophilic alcohols are potent complement activators. Very hydrophilic, poorly nucleophilic, and H-bond accepting polymers (=‘stealth’ polymers) have a better performance: Dextran [55] or poly(N-vinylpyrrolidone) (PVP) [56] are moderate to weak activators, and polyoxazolines [57], PEG [58] (but not necessarily its block copolymers, such as Pluronics [25]), and phosphoryl choline-based macromolecules [59] are low- to non-activating.

Here, we compared the capacity of mPPSO-OH to produce C3a and C5a anaphylatoxins (the small, soluble fragments of complement C3 and C5 proteins liberated upon cleavage by their respective C3 or C5 convertase) from human plasma with those of mPEG-OH and mono-hydroxy poly(2-methyl oxazoline) (PMOX-OH), using zymosan as a positive control (Figure 3A,B). Please note that the three synthetic polymers are all of similar size (around 5 kDa) and all feature one terminal OH group. At any of the three concentrations tested, complement activation followed the trend: Zymosan >> mPEG-OH ≥ PMOX-OH ≥ mPPSO-OH. In a direct comparison to mPEG-OH, at a concentration of 0.8 mg/mL mPPSO-OH produced 30% less C3a and 34% less C5a than mPEG-OH. As a caveat, an in vitro low complement activation does not imply the absence of complement-related issues in vivo; for example, complement plays a major role in the initiation of ABC observed with theoretically low activators, such as PEGylated, and probably also with PMOXylated nano-carriers.

Finally, the low uptake of PPSO by activated macrophages also suggests a ‘stealth’ behaviour. Murine RAW264.7 macrophages were activated with LPS and exposed for 24 h to various concentrations of FITC-labelled PPSO, PEG, or cationic dextran, which was used as a positive control; cell lysates were then used to quantify the polymer uptake, including in it both the material internalized and that bound on cell surfaces.

At any of the four concentrations tested, the 24-h uptake varied in the order, cationic dextran >> PEG ≥ PPSO (Figure 3C, left); this trend becomes even more apparent when looking at the uptake efficiency (% of polymer taken by cells; Figure 3C, right). In short, PPSO appears to resist both binding and internalization in activated macrophages similarly, if not marginally better than PEG. This general behaviour is likely ascribed to the same substantial lack of interactions between PEG or PPSO and biomolecules, with internalization probably only occurring through non-receptor-mediated mechanisms, e.g., macropinocytosis. Although the PPSO/PEG difference is not statistically significant in all cases, the uptake of the PPSO is typically lower; this might be caused by partial de-activation of the macrophages as a result of ROS scavenging, which we previously invoked to explain the slightly higher toxicity of PPSO. To that end, it is therefore necessary to demonstrate that PPSO is indeed both ROS scavenging and anti-inflammatory.

**Figure 4:**
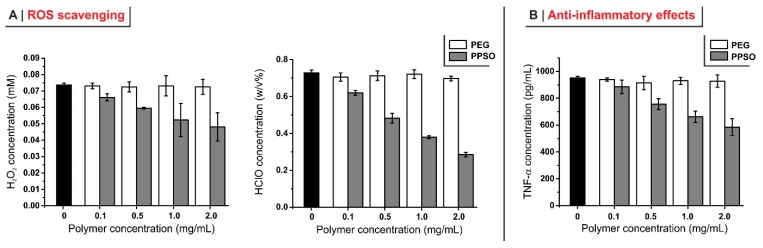
(**A**) Concentration of H_2_O_2_ (left) and hypochlorite (right) in cell lysates after 24 h exposure of LPS-activated macrophages to mPEG-OH and mPPSO-OH. Please note that although hypochlorite is more commonly associated to the neutrophil respiratory burst, it is also routinely produced by LPS stimulated macrophages [60]. (**B**) The anti-inflammatory activity of PPSO was evaluated by measuring the TNF-α levels in the supernatant of RAW264.7 macrophages stimulated with LPS and exposed for 24 h to mPPSO-OH or mPEG-OH.

To do that, we measured the concentration of two of the most important biological ROS (H_2_O_2_ and ClO^−^) in cell lysates, thereby accounting for both intra- and extracellular ROS. PPSO reduced the levels of both ROS in a dose-dependent fashion, whereas at no concentration did PEG appear to have an influence on their levels (Figure 4A). This differential PEG/PPSO behaviour was also recorded on the extracellular levels of TNF-α, whose dose-dependent reduction with PPSO concentration is ascribed to ROS scavenging (Figure 4B, as we have seen, e.g., in vitro and in vivo with PPS (polysulfide) nanoparticles) [44].

## 3. Materials and Methods

### 3.1. Materials, (Bio)Chemicals, and Cells

All chemicals were used as received from suppliers unless otherwise stated. S-methyl thioacetate, sodium methoxide (MeONa, 0.5 M in methanol), tributyl phosphine,1,8-diazabicyclo[5.4.0]undec-7-ene (DBU), sodium sulfate anhydrous (Na_2_SO_4_), dichloromethane, tetrahydrofuran (THF), N,N-dimethyl formamide (DMF), methanol, and hydrochloric acid 1 M (HCl) were purchased from Thermo Fisher Scientific (Loughborough, UK). HPLC grade DMF was purchased from Alfa Aesar/Thermo Frsher. Trifluoroacetic acid (TFA), poly(ethylene glycol) monomethyl ether (mPEG-OH) (5 kDa), and mono hydroxy-terminated poly(2-methyl-2-oxazoline) (PMOX-OH) were purchased from Sigma-Aldrich (Gillingham, UK). Phosphate buffered saline (PBS) Dulbecco A tablets were purchased from Oxoid (Basingstoke, UK). Further, 30%wt. H_2_O_2_ was purchased from VWR (Leicester, UK) and titrated to determine its actual concentration prior to use. C3a, C5a Platinum ELISA kits were purchased from eBioscience (San Diego, CA, USA).

Human dermal fibroblasts (neonatal) (HDFn) and lipopolysaccharides (LPS) from *Escherichia coli* 026:B6 were purchased from Thermo Fisher Scientific; RAW264.7 Macrophages were purchased from ATCC. Citrated human plasma, fluorescein isothiocyanate (FITC), and FITC–labelled cationic dextran (nominal molecular weight of 4 kDa) were purchased from Sigma Aldrich. Dulbecco’s modified Eagle’s medium (DMEM), fetal bovine serum (FBS), L-glutamine 200 mM (×100), and penicillin-streptomycin were purchased from Invitrogen (Paisley, UK). The MTS proliferation assay (CellTiter 96^®^ Aqueous One Solution Cell Proliferation Assay) was purchased from Promega (Madison, WI, USA). The ELISA kit for TNFα was purchased from Cohesion Biosciences (London, UK). The Hypochlorite Assay Kit (colorimetric, absorbance at 555 nm) and Hydrogen Peroxide Assay Kit (colorimetric/fluorometric; exc. 535 nm, em. 587 nm) were purchased from MitoSciences, Abcam^®^ (Cambridge, UK).

### 3.2. Physico-Chemical Characterization

^1^H NMR spectra were recorded on 1.5% wt. solutions in deuterated chloroform and dimethyl sulfoxide (*d6*-DMSO) using a Bruker Avance 300 or 400 MHz Bruker spectrometer.

FT-IR spectra were recorded in ATR mode (Golden Gate) on a Tensor 27 Bruker spectrometer (Bruker UK Limited, UK) equipped with a 3000 Series TM High Stability Temperature Controller (Specac, UK).

Gel permeation chromatography (GPC) analysis was performed using triple detection conditions with a Malvern OmniSec System (Malvern, UK) comprising of a D6000M and D3000 column in sequence and operating online at 50 °C with HPLC grade DMF containing 0.1% LiBr as the mobile phase at a flow rate of 1.0 mL/min. Calibration was performed using a poly(methyl methacrylate) standard of a known molecular weight, intrinsic viscosity, and dn/dc.

UV-Vis and fluorescence readings were obtained through a BioTek Synergy 2 multimode microplate reader (BioTek, UK) at a temperature of 37 °C.

### 3.3. Preparative Operations

#### 3.3.1. S-(2-((Tetrahydro-2H-pyran-2-yl)oxy)ethyl) ethanethioate (**1**)

In total, 6.0 g (36.4 mmol) of 2-(2-chloroethoxy)tetrahydro-2H-pyran and 6.24 g (54.67 mmol, 1.5 equiv.s) of potassium thioacetate were added under argon to 100 mL of acetonitrile in a three-necked rounded-bottom flask. The mixture was added to a heating bath at 45 °C and allowed to stir overnight (~12 h). Volatiles were removed by rotary evaporation and the viscous oil was dissolved into 80 mL of diethyl ether and extracted against 20 mL of a 5% sodium bicarbonate solution (×3) then brine (2×). The organic phase was separated, dried over Na_2_SO_4_, filtered, and concentrated in vacuo. The resulting oil was purified by column chromatography using silica as the stationary phase and 9:1 hexane:ethyl acetate as the mobile phase to yield 6.0 g of faintly yellow oil (81% yield).

^1^H NMR (CDCl_3_): δ = 1.46–1.91 (m, 6H, -OCH-[(C**H_2_**)_3_-CH_2_-O-]), 2.35 (s, 3H, -S-C(=O)-C**H_3_**), 3.09–3.18 (t, 2H, -O-CH_2_-C**H_2_**-S-), 3.46–3.61 (m, 2H, -OCH-[(C**H_2_**)_3_-CH_2_-O-]), 3.78–3.92 (m, 2H, -O-C**H_2_**-CH_2_-S-), 4.60–4.66 ppm (t, 1H, -OC**H**-[(CH_2_)_3_-CH_2_-O-]).

#### 3.3.2. Monohydroxy-Terminated Poly(Propylene Sulfide) (PPS)_56_

In total, 246 mg (1.20 mmol) of S-(2-((tetrahydro-2H-pyran-2-yl)oxy)ethyl) ethanethioate, 10.89 mL (3.61 mmol, 3 equiv.s) of tributylphosphine and then 2.53 mL (1.26 mmol, 1.05 equiv.s) of a 0.5 M sodium methoxide in methanol solution were added under an argon atmosphere to 12 mL of degassed THF (bubbled with argon for 45 min). After 10 min, 5.0 g (67.43 mmol, 56 equiv.s) of PS were added and allowed to react for a further 75 min. Next, 342 mg (2.41 mmol, 2 equiv.s) of iodomethane were added and allowed to react for 60 min; excess iodomethane was quenched by 1.2 mL (2.41 mmol, 2 equiv.s) of a 2 M dimethylamine in THF solution. Volatiles were removed in a Genevac EZ2 Elite centrifugal evaporator and the resulting oil was dissolved in 40 mL of dichloromethane and extract 5 times against 10 mL of brine. The organic phase was separated, dried over Na_2_SO_4_, filtered and concentrated in vacuo. The concentrated oil was then precipitated into 8 mL of MeOH, centrifuged, and the MeOH phase decanted. The pellet was washed with 5 mL of MeOH a further 3 times and dried in vacuo to yield colourless viscous oil. Please note that the initiator was probably partially deprotected, most likely as a result of HI formed during iodomethane quenching.

Deprotection. In total, 2.0 g of PPS_56_ (0.47 mmol) were dissolved into 5 mL of a 1:1 methanol/THF mixture, to which 3 drops of water were added before being transferred to a reaction flask containing 1.4 g of acidic ion exchange resin Dowex 50W X8 (70 wt. % of PPS_56_ weight), which was activated with 1.0 M HCl prior to the reaction. The mixture was then heated to 50 °C and allowed to react for 24 h (no stirring). Resin was filtered out and the volatiles were removed under reduced pressure to produce colourless viscous oil.

^1^H NMR (CDCl_3_): δ = 1.30–1.45 (d, C**H_3_** in PPS chain), 2.13 (s, 3H, -CH(CH_3_)-S-**CH_3_**, terminal methyl), 2.56–2.71 (m, PPS chain: 1 diastereotopic C**H_2_**), 2.74–2.80 (t, 2H, HO-CH_2_-C**H_2_**-S-), 2.81–3.05 (m, C**H** and 1 diastereotopic H of CH_2_ in PPS chain), 3.72–3.79 ppm (t, 2H, HO-C**H_2_**-CH_2_-S-).

ATR FT-IR (thin film): 2959 (ν_s_ CH_3_), 2918 (ν_as_ CH_2_), 2865 (ν_as_ CH_3_ and ν_s_ CH_2_), 1450 (δ_s_ CH_2_), 1371 (δ_s_ CH_3_), 1171 (CH_2_ wag), 734 (ρ CH_2_), and 689 cm^−1^ (ν C−S).

#### 3.3.3. Poly(Propylene Sulfoxide) mPPSO-OH and Poly(Propylene Sulfone) mPPSO_2_-OH

In total, 1.75 g of PPS (~23 mmol of thioethers) and 180 mg of diphenyl diselenide (DPDS) (0.58 mmol, ~0.025 equiv.s per thioethers) were dissolved in 25 mL of DMF. Either 2.6 mL (25.45 mmol, 1.1 equiv.s per thioether) or 5.2 mL (50.90 mmol, 2.2 equiv.s per thioether) of a 30% (w/w) H_2_O_2_ aqueous solution were then added and allowed to react for 24 hours for the PPSO or PPSO_2_ syntheses respectively. Note, upon addition of the aqueous H_2_O_2_, some precipitation/cloudiness of PPS occurred, and a small amount of acetone was added to give a clear solution. The solution was evaporated using a Genevac EZ2 Elite centrifugal evaporator and the resulting oil was dissolved into a small amount of dichloromethane and precipitated into diethyl ether (5 times). The resulting pellet was dried in a vacuum oven at 40 °C for 24 h to give a white solid.

*PPSO*. ^1^H NMR (CDCl3): δ = 1.29–1.66 (C**H_3_** in PPSO chain), 3.09 (s, 3H, -CH(CH_3_)-S(=O)_2_-C**H_3_**, terminal methyl), 2.61–3.01 (HO-CH_2_-C**H_2_**-S(=O)- and PPSO chain: 1 diastereotopic C**H_2_**), 3.14–3.62 ppm (m, C**H** and 1 diastereotopic H of C**H_2_** in PPSO chain), 3.72–3.79 (t, 2H, HO-C**H_2_**-CH_2_-S(=O)-). ATR FT-IR (thin film): 2965 (ν_s_ CH_3_), 2916 (ν_as_ CH_2_) 2869 (ν_as_ CH_3_ and ν_s_ CH_2_), 1453 (δ_s_ CH_2_), 1377 (δ_s_ CH_3_), 1017 (ν_s_ S=O), 834, 726 (ρ CH_2_), 646 cm^−1^ (ν C−S).

*PPSO*_2_. ^1^H NMR (*d6*-DMSO): δ = 1.35–1.61 (C**H_3_** in PPSO_2_ chain), 3.09 (s, 3H, -CH (CH_3_)-S (=O)_2_-C**H_3_**, terminal methyl) 3.77 (2H, HO-C**H_2_**-CH_2_-S(=O)_2_- under H_2_O signal), 3.44–3.70 (PPSO_2_ chain: 1 diastereotopic C**H_2_**), 3.77–4.14 ppm (m, C**H** and 1 diastereotopic H of C**H_2_** in PPSO chain). ATR FT-IR (thin film): 2985 (ν_s_ CH_3_), 2939 (ν_as_ CH_2_) 1663 (ν_s_ C=O), 1453 (δ_s_ CH_2_), 1377 (δ_s_ CH_3_), 1296 (ν_as_ S (=O)_2_) 1121 (ν_s_ S(=O)_2_), 844 (ν S-O), 742 cm^−1^ (ρ CH_2_).

#### 3.3.4. FITC-Labelled Polymers

PEG-amine was synthesized from mPEG-OH using a Mitsunobu reaction as previously described [61]. The synthesis of PPSO-amine is provided in the Supplementary Materials, Section 1SI. In total, 2 mL of degassed dry DMF (argon bubbling for 45 min) were transferred in each position of the reactor with 100 mg (0.02 mmol) of mPEG-amine or PPSO-amine followed first by 8 mg (0.08 mmol, 4 equiv.s) of triethylamine (TEA) and then 7.8 mg (0.02 mmol, 1 equiv.s) of FITC in dry DMF (note: Weighing vials were washed after weighting with an additional 100 uL of DMF, which was added into the reaction mixture). The mixture was allowed to react for 24 h at room temperature. Subsequently, the solution was evaporated using a Genevac EZ2 Elite centrifugal evaporator and the resulting product was dissolved into a small amount of water and dialysed against deionized water for several days, until free dye molecules were completely removed (no fluorescence detected in dialysate).

### 3.4. Biochemical/Biological Assays

#### 3.4.1. Cell Viability

The cytotoxicity of mPPSO-OH was assessed on HDFn using mPEG-OH as a control and employing the 3-(4,5-dimethylthiazol-2-yl)-5-(3-carboxymethoxyphenyl)-2-(4-sulfophenyl)-2H-tetrazolium (MTS) assay of mitochondrial reductase activity. HDFs were plated into 96-well plates at a density of 10,000 cells/well, allowing the cells to adhere by incubating them in DMEM complete medium (10% FBS) for 24 h. In total, 100 µL of 0.01, 0.05, 0.1, 0.5, 1, 5, and 10 mg/mL polymer solutions in the culture medium were introduced into wells containing 100 µL of medium. After 24 or 48 h of incubation at 37 °C, the medium was discarded, cells were gently washed with PBS, and 120 µL of MTS solution (Cell Titer 96 Aqueous One Solution Reagent in medium prepared following the manufacturer instructions) were added to each well, incubating the plates for 1 h at 37 °C. Formazan absorbance was measured at 490 nm and expressed as a percentage of the untreated controls.

#### 3.4.2. Complement Activation

The cleavage of complement components C3 and C5 was monitored by measuring the formation of their activation products, C3a and C5a, using a commercial C3a and C5a ELISA kit (Platinum eBioscience kit, Invitrogen, Carlsbad, CA, USA). Activation studies were performed by mixing 450 µL of human plasma with 150 µL of polymer-PBS solutions (mPPSO-OH, PMOX-OH and mPEG-OH) at concentrations of 0.8, 0.4, or 0.1 mg/mL. The solutions were then incubated in a shaking water bath at 37 °C for 30 min. Reactions were terminated by addition of the “sample diluent” provided with the assay kit. Control plasma incubations contained phosphate buffered saline (the same volume as the polymer solutions) for assessing background levels of complement activation products. Zymosan (Sigma-Aldrich, St. Louis, MO, USA) was used as a positive control for complement activation. Briefly, the samples were diluted with the dilution buffer provided in the kit and added to a microtiter plate coated with a monoclonal antibody specific for human C3a and C5a. After 1 h of incubation at room temperature to allow for C3a and C5a binding, the plates were washed and incubated for an additional hour with peroxidase–conjugated rabbit anti-C3a, and anti-C5a. Following a final washing step, the chromogenic substrate was added to detect bound C3a, and C5a, recording the absorbance at 450 nm. The values reported are means +/− standard deviations, for *n* = 3 and reported as mass of C3a or C5a per mL of plasma per well.

#### 3.4.3. Polymer Uptake

RAW 264.7 macrophages were seeded in black 96-well flat-bottom microtiter plates at a concentration of 2.5 × 10^4^ cells per well and kept at 37 °C, in a 5% CO_2_ incubator overnight in DMEM complete medium. Subsequently, the cells were washed once with PBS. Fluorescently labelled PPSO, cationic dextran, and PEG at a concentration of 0.1, 0.5, 1, and 2 mg/mL were added into each well in 100 µL of complete medium and incubated for 24 h. For LPS activation, 24 h after seeding the cells, they were incubated for an additional 24 h with a 500 ng/mL LPS solution in DMEM complete medium. Subsequently, 500 ng/mL of LPS together with fluorescently labelled polymers at concentrations of 0.1, 0.5, 1, and 2 mg/mL were added into each well.

Cells were then washed 3 times with PBS and a 0.5% solution of Triton X-100 in 0.2 N sodium hydroxide was added for 10 min at 37 °C to lyse the cells. The fluorescence of the cell lysates was measured using excitation and emission filters respectively at 485 ± 20 nm and 528 ± 20 nm and a calibration with dispersions of fluorescently labelled polymers in cell lysates with polymer concentration ranging from 0.05 to 12.5 µg/mL.

#### 3.4.4. ROS-Scavenging Effects of PPSO (Analysis of H_2_O_2_ and ClO^−^)

RAW264.7 macrophages were plated in transparent 96-well flat-bottomed plates at a concentration of 2.5 × 10^4^ cells per well and kept at 37 °C, in a 5% CO_2_ incubator overnight in DMEM complete medium; the cells were then washed once with PBS and treated for 24 h with 500 ng/mL LPS alone or with mPEG-OH or mPPSO-OH at concentrations of 0.1, 0.5, 1, 2, and 4 mg/mL in DMEM complete medium. The production of H_2_O_2_ and hypochlorite anion (ClO^−^) was assessed on cell lysates (see above) using the Hydrogen Peroxide (colorimetric/fluorometric) and Hypochlorite Assay Kits (colorimetric), respectively. Both kits were used in accordance with the manufacturer’s instructions.

#### 3.4.5. Anti-Inflammatory Effect of PPSO (Analysis of TNF-α)

RAW264.7 macrophages were seeded in 24-well flat-bottomed plates at a concentration of 1 × 10^5^ cells per well and kept at 37 °C, in a 5% CO_2_ incubator overnight. The cells were then washed once with PBS. In total, 500 µL of a solution containing 500 ng/mL LPS alone or with polymers at concentrations of 0.1, 0.5, 1, and 2 mg/mL in DMEM complete medium were added into each well. Following a 24-h incubation period, the supernatants were separated and centrifuged at 13,000 rpm for 5 min and were kept at −80 °C until the ELISA analysis of TNF-α was performed, using the Cohesion Bioscience Human TNFα ELISA (Enzyme-Linked Immunosorbent Assay) kit. This assay employs 96-well plates coated with an antibody specific for human TNFα, and an HRP-based colorimetric detection (absorbance at 450 nm).

## 4. Conclusions

In this study we provided a-proof-of-principle that sulfoxide-based polymers may present both ‘stealth’ and responsive/pharmacologically active (‘drug’-like) features, with clear ROS scavenging activity at least at concentrations above 0.1 mg/mL. Clearly, more detailed investigations on PPSO are required in order to, e.g., ascertain the mechanism of endocytosis, efficiency of ROS removal, interactions with proteins, pharmacokinetics, and in vivo effects on inflammation models, etc. Nevertheless, we believe this paper to be the first report of a ‘stealth’ polymer that by virtue only of its own main chain—and not of a more or less complex (bio)functionalization—may both be considered on the one hand biocompatible and inert, yet on the other hand, biologically active and anti-inflammatory.

## Figures and Tables

**Table 1:** Molecular weight data of mPPS-OH and mPPSO-OH.

	$\bar{M_{n}}$ (g/mol)/$\bar{DP_{n}}$			Đ ^b^	End-Capping Yield (mol %) ^c^
	Theor.	^1^H NMR ^a^	GPC ^b^		
mPPS_56_-OH	4315/56	4390/57	3850/≈49	1.26	98
mPPSO_56_-OH	5211/56	5300/57	4400/≈47	1.21	not applicable

^a^$\bar{DP_{n}}$ obtained from the comparison of the resonance of primary alcohol’s CH_2_ group at ~3.75 ppm with the main chain methyl peak of PPS at ~1.33 to 1.45 ppm (resonances *a* and *e* in the CDCl_3_ spectrum of deprotected mPPS-OH; see Supplementary Materials, Figure 1SI). $\bar{M_{n}}$ calculated from $\bar{DP_{n}}$ ^b^ From triple detection GPC in DMF containing 0.1% LiBr. ^c^ From the comparison of the ^1^H NMR resonances of the two terminal groups (resonances *a* and *f* in the CDCl_3_ spectrum of deprotected PPS; Supplementary Materials, Figure 1SI).

## Supplementary Materials

Supplementary materials can be found at https://www.mdpi.com/1422-0067/20/18/4583/s1.
