# Supplementary material for: Main Chain Polysulfoxides as Active ‘Stealth’ Polymers with Additional Antioxidant and Anti-Inflammatory Behaviour
Source: Int J Mol Sci. 2019 Sep 17;20(18):4583. doi: 10.3390/ijms20184583 (PMC6770853; doi:10.3390/ijms20184583)
Supplement: Supplementary file 1 [file ijms-20-04583-s001.pdf]

Supplementary Material

1. SI Additional experimental descriptions

Synthesis of PPSO-amine

PPS-Boc. 183 mg of S-phenyl thioacetate (1.20 mmol) was added under an argon atmosphere to 12 mL of degassed THF (bubbled with argon for 45 minutes) followed by 1.8 mL of tributylphosphine (7.226 mmol, 6 equiv.s) and then 2.53 mL (1.26 mmol, 1.05 equiv.s) of a 0.5 M sodium methoxide in methanol solution. After 10 minutes, 5.0 g (67.44 mmol, 56 equiv.s) of PS was added and allowed to react for further 75 minutes. 1.1 g of N-Boc-2-chloroethylamine (6.02 mmol, 5 equiv.s) was added to end-cap the thiolate and allowed to react for 60 minutes. Volatiles were removed in a Genevac EZ2 Elite centrifugal evaporator and the resulting oil was dissolved in 40 mL of dichloromethane and extract 5 times against 10 mL of brine. The organic phase was separated, dried over Na_2SO_4, filtered and concentrated in vacuo. The concentrated oil was then precipitated into 8 mL of MeOH, centrifuged and the MeOH phase decanted. The pellet was washed with 5 mL of MeOH a further 3 times and dried *in vacuo* to yield colourless viscous oil.

^{1}H NMR (DMSO): δ = 1.21-1.33 (d, C**H**$_3$ in PPS chain), 1.38 (s, 9H, -C(=O)-O-C(-C**H**$_3$)$_3$, terminal methyls), 2.54-2.69 (m, -C(CH$_3$)-S-C**H**$_2$- and PPS chain: 1 diastereotopic H of C**H**$_2$), 2.81-3.02 (m, C**H** and 1 diastereotopic H of C**H**$_2$ in PPS chain), 4.07-4.13 (m, 2H, S-CH$_2$-C**H**$_2$-), 7.15-7.40 (m, 5H of phenyl).

^{13}C NMR (CDCl$_3$): δ = 20.75 (**C**H$_3$ in PPS chain), 28.43 (-S-**C**H$_2$-CH$_2$-NH- and -O-C-(**C**H$_3$)$_3$, terminal methyl of boc group), 38.38 (**C**H in PPS chain), 40.18 (-S-CH$_2$-**C**H$_2$-NH-), 41.22 (**C**H$_2$ in PPS chain), 79.43 (-O-**C**-(CH$_3$)$_3$), 126.45, 129.11, 129.87, 136.03 (phenylic carbons), 155.75 (CH$_2$-NH-**C**(=O)-O-).

FT-IR (cm^{-1}): 2962 (ν_s CH$_3$), 2921 (ν_{as} CH$_2$ of CH$_2$-S), 1711 (ν_{as} C=O), 1449 (ν_{as} C=C of phenyl), 1374 (δ_s CH$_3$), 1171 (CH$_2$ wag), 737 (ϱ CH$_2$), 688 (ν C–S).

PPSO-Boc. 1.7 g (0.4 mmol, ~ 23.1 mmol of thioether) of PPS and 180 mg (0.6 mmol, 0.025 equiv.s per thioether) of diphenyl diselenide (DPDS) were dissolved in 25 mL of DMF. Either 2.6 mL (25.4 mmol, 1.1 equiv.s per thioether) of a 30% (w/w) H_2O_2 aqueous solution were then added and allowed to react for 24 hours for the PPSO synthesis. Note, upon addition of the H_2O_2, some precipitation/cloudiness occurred and a small amount of acetone was added to give a clear solution. The solution was evaporated using a Genevac EZ2 Elite centrifugal evaporator and the resulting oil was dissolved into a small amount of dichloromethane and precipitated into diethyl ether (5 times). The resulting pellet was dried in a vacuum oven at 40°C for 24 hours to give a white solid.

PPSO amine. 1.0 g of PPSO-Boc (0.2 mmol) were dissolved into 10 mL of dichloromethane, to which 114 mg of TFA was added and allowed to react for 24 hours at room temperature. The solution was then precipitated into 50 mL of toluene (5 times). Volatiles were removed in a Genevac EZ2 Elite centrifugal evaporator.

^{1}H NMR (DMSO): δ = 1.29-1.43 (d, C**H**$_3$ in PPSO chain), 2.75-3.05 (m, C**H** in PPSO chain), 3.05-3.35 (m, 1 diastereotopic H of C**H**$_2$ in PPSO chain and -C(CH$_3$)-S(O)-C**H**$_2$-), 3.35-3.42 (t, 2H, S-CH$_2$-C**H**$_2$-NH$_2$), 3.51-3.62 (s, 1 diastereotopic H of C**H**$_2$ in PPSO chain), 7.46-7.99 (m, 5H in phenyl)

FT-IR (cm^{-1}): 3430 (ν_s NH$_2$), 2973 (ν_s CH$_3$), 2925 (ν_{as} CH$_2$ of CH$_2$-S), 1639 (δ_s NH$_2$), 1452 (ν_{as} C=C of phenyl), 1306 (δ_s CH$_3$), 1130 (CH$_2$ wag), 1018 (ν of S=O).

2. SI Additional figures

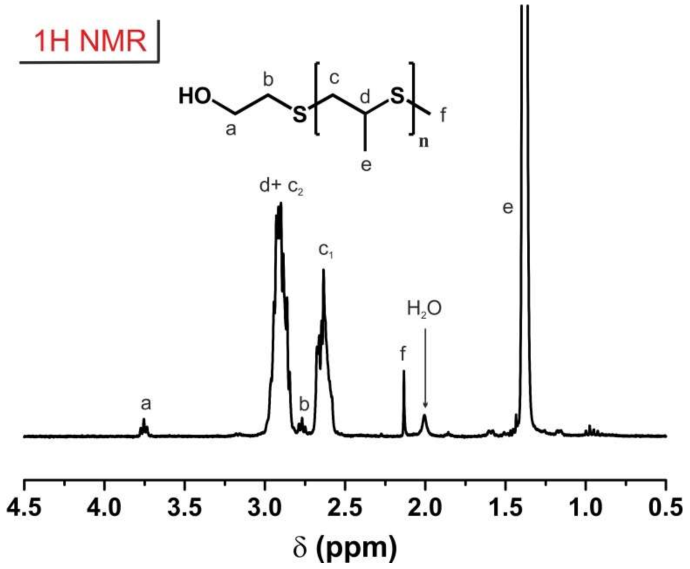

Figure 1SI. ^{1}H NMR of primary alcohol-terminated PPS_{56} in $CDCl_3$.

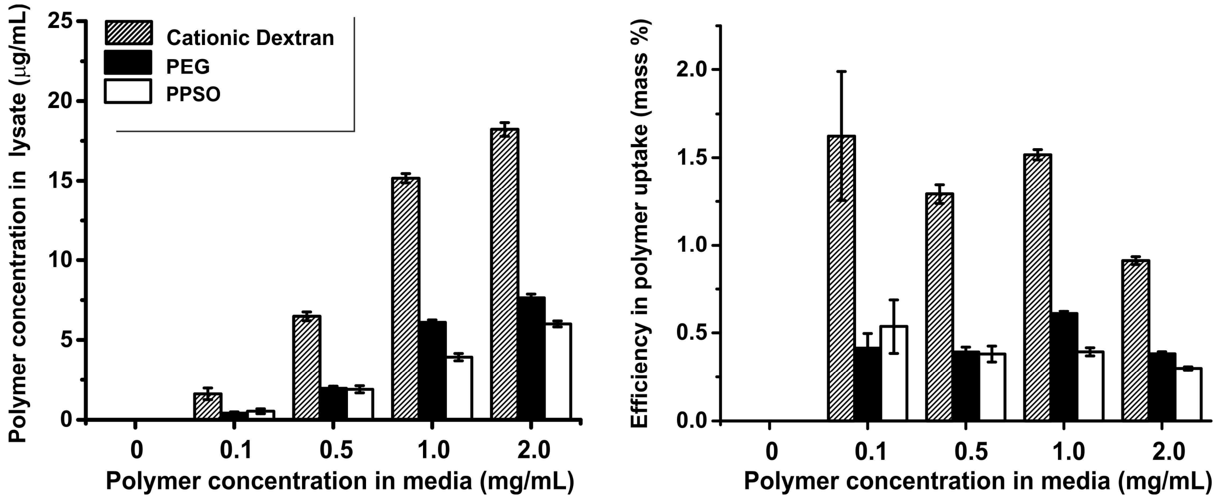

Figure 2SI. Uptake of fluorescein-labelled PEG and PPSO (the terminal OH groups were converted to amines and reacted with FITC) and FITC-labelled cationic dextran after 24 h incubation in RAW macrophages.
